# L-Carnosine Affects the Growth of *Saccharomyces cerevisiae* in a Metabolism-Dependent Manner

**DOI:** 10.1371/journal.pone.0045006

**Published:** 2012-09-12

**Authors:** Stephanie P. Cartwright, Roslyn M. Bill, Alan R. Hipkiss

**Affiliations:** Aston Research Centre for Healthy Ageing, School of Health and Life Sciences, Aston University, Birmingham, United Kingdom; Texas A&M University, United States of America

## Abstract

The dipeptide L-carnosine (β-alanyl-L-histidine) has been described as enigmatic: it inhibits growth of cancer cells but delays senescence in cultured human fibroblasts and extends the lifespan of male fruit flies. In an attempt to understand these observations, the effects of L-carnosine on the model eukaryote, *Saccharomyces cerevisiae*, were examined on account of its unique metabolic properties; *S. cerevisiae* can respire aerobically, but like some tumor cells, it can also exhibit a metabolism in which aerobic respiration is down regulated. L-Carnosine exhibited both inhibitory and stimulatory effects on yeast cells, dependent upon the carbon source in the growth medium. When yeast cells were not reliant on oxidative phosphorylation for energy generation (e.g. when grown on a fermentable carbon source such as 2% glucose), 10–30 mM L-carnosine slowed growth rates in a dose-dependent manner and increased cell death by up to 17%. In contrast, in media containing a non-fermentable carbon source in which yeast are dependent on aerobic respiration (e.g. 2% glycerol), L-carnosine did not provoke cell death. This latter observation was confirmed in the respiratory yeast, *Pichia pastoris*. Moreover, when deletion strains in the yeast nutrient-sensing pathway were treated with L-carnosine, the cells showed resistance to its inhibitory effects. These findings suggest that L-carnosine affects cells in a metabolism-dependent manner and provide a rationale for its effects on different cell types.

## Introduction

The dipeptide L-carnosine (β-alanyl-L-histidine) was characterized more than a century ago and occurs naturally in tissues such as brain, kidney and skeletal muscle at concentrations up to 20 mmol kg^−1^ dry muscle mass [1]. While related dipeptides such as anserine and homocarnosine are also found in the muscle tissues of mammals, birds and fish [2], they do not seem to exhibit the range of properties shown by L-carnosine [3], which may be a consequence of insufficient study (but see reference [7] for a comparison with L-carnosine). These include action as a hydrogen ion buffer, neurotransmitter, anti-oxidant, anti-glycator, metal ion scavenger, immuno-stimulant and wound healing agent [4], [5]. Moreover, L-carnosine can delay cell senescence and reverse the senescent appearance of cultured human fibroblasts [3], as well as extending lifespan in *Drosophila melanogaster* [6]. In contrast, L-carnosine selectively inhibits the growth of transformed cell lines [7] and other tumor cells [8], most likely by inhibiting cellular ATP generation [9], [10].

The unusual metabolism of tumor cells, in which metabolic flux is directed away from oxidative phosphorylation, is considered a possible target for developing novel anti-cancer therapies [11], [12]: tumor cells can adapt to have a decreased dependence on oxidative phosphorylation for energy generation compared with normal cells, even in the presence of adequate oxygen. This is known as the Warburg effect [13]. The Crabtree effect [14], the inhibition of respiration in the presence of glucose, is also a key metabolic feature of tumors, rapidly-proliferating normal cells and the respiro-fermentative yeast species, *Saccharomyces cerevisiae*: when grown on a fermentable carbon source, such as 2% glucose, aerobic respiration is actively down-regulated in this yeast [15], [16]. Consequently, both tumor cells and fermenting yeast derive the majority of their ATP production from glycolysis. Notably, *S. cerevisiae* can also grow on non-fermentable carbon sources, such as 2% glycerol, by employing oxidative phosphorylation for ATP generation.

Given the contrasting actions of L-carnosine on different cell types, the dipeptide has been described as enigmatic [17]. In an attempt to understand whether these observations might be due to a cell’s metabolic characteristics, *S. cerevisiae* was chosen as a test system on account of its unique metabolic properties. Here we show that L-carnosine slows growth rates and increases cell death of yeast cells when they are reliant on glycolysis for energy generation. When yeast is reliant on oxidative phosphorylation, it is resistant to L-carnosine’s inhibitory effects. These metabolism-dependent findings provide a rationale for the enigmatic effects displayed by L-carnosine.

## Materials and Methods

### Yeast Strains and Culturing Conditions

*S. cerevisiae* BY4741 is the parental strain of the deletion mutants *tor1*Δ, *tpk1*Δ, *gpa2*Δ, *gpr1*Δ, *hxk2*Δ, *rpl31a*Δ and *sch9*Δ (from the EUROSCARF collection: http://web.uni-frankfurt.de/fb15/mikro/euroscarf), and as such provided the wild-type control. Yeast cells were cultured in YP or 2×CBS with the desired carbon source added to 2% w/v. YP contained 1% yeast extract, 2% bacto peptone and 2% of the desired carbon source (glucose, glycerol, mannose, galactose, fructose, xylose, sorbitol or ribose). 2×CBS was composed of 10 g/L ammonium sulfate, 6 g/L potassium dihydrogen phosphate, 1 g/L magnesium sulfate heptahydrate supplemented with 2% glucose, 2×DO solution, 100 mM MES, 2 mL/L each of trace element solution and vitamin stock solution. 10×DO solution (per L) was composed of 200 mg adenine hemisulfate, 200 mg l-arginine hydrochloride, 200 mg l-histidine hydrochloride monohydrate, 300 mg l-isoleucine, 1,000 mg l-leucine, 300 mg l-lysine hydrochloride, 200 mg l-methionine, 500 mg l-phenylalanine, 2,000 mg l-threonine, 200 mg l-tryptophan, 300 mg l-tyrosine, 200 mg uracil, 1,500 mg l-valine. 250 mL trace element solution was composed of 3.75 g EDTA, 1.125 g zinc sulfate heptahydrate, 0.25 g magnesium chloride tetrahydrate, 0.075 g colbalt (ΙΙ) chloride hexahydrate, 0.075 g copper (ΙΙ) sulfate pentahydrate, 0.1 g sodium molybdenum dehydrate, 1.125 g calcium chloride dehydrate, 0.75 g iron (II) sulfate heptahydrate, 0.25 g boric acid and 0.025 g potassium iodide. 250 mL vitamin stock solution was composed of 0.0125 g biotin, 0.25 g calcium-d-pantothenate, 0.25 g nicotinic acid, 6.25 g myo-inositol, 0.25 g thiamine hydrochloride, 0.25 g pyridoxine hydrochloride and 0.05 g d-amino benzoic acid; pH was maintained at 6.5. *P. pastoris* strain X33 was cultured in YP with glucose added to 2% w/v. To initiate an experiment, 5 mL YP or 2×CBS supplemented with 2% glucose were inoculated with a single yeast colony, grown to logarithmic phase and used to inoculate a 125 mL baffled shake-flask containing 25 mL growth medium. Cultures were incubated at 30°C, 220 rpm. Stock solutions of 200 mM L-carnosine, β-alanine, L-histidine (Molekula, Dorset, UK) and D-carnosine (the generous gift of Flamma Chemicals, Milan, Italy) were prepared with double distilled water, the pH was adjusted to 6 and the solutions filter sterilized prior to use.

### Sampling and Extracellular Substrate Determination

Samples were withdrawn at various time points to determine OD_600_, residual glucose concentration, ethanol concentration and cell viability. 1 mL culture was centrifuged at 5,000 *g*, 4°C for 5 min. 0.5 mL of the supernatant was stored at –20°C for glucose and ethanol analyses. Glucose concentrations were calculated with an Accu-Chek Active glucose analyzer (Roche Diagnostics, UK). Ethanol analysis was performed by gas chromatography using a CSi 200 series gas chromatograph. To determine cell viability, 2 µL culture was added to a microscope slide with 2 µL trypan blue solution; 100 cells were counted at 100× magnification and the number of stained cells determined.

### On-line Flow Microcalorimetry

Flow microcalorimetric data were collected for shake-flask cultures at 30°C, 220 rpm, using a Thermal Activity Monitor 2277 with a flow vessel working volume of 0.6 cm^3^ (Thermometric AB, Sweden) as previously described [18]. Briefly, the system was sterilized by successively pumping through solutions of sterile deionized water, 70% ethanol (v/v), sodium hydroxide (0.5 M) and finally sterile deionized water, then allowing the flow lines to run dry before inserting into a culture. The pump rate was 20 rpm using a Watson Marlow 400 pump. The thermostatic water bath was maintained at 30°C. The software used to record data was Digitam v4.1. The calorimeter was calibrated at the same temperature, flow rate and amplifier setting (1,000 µW) to be used during experimental data collection in order to establish a steady baseline deflection. Electrical calibration was done in the culture medium. Once the baseline was established, the outsides of the inlet and outlet tubes were sterilized with 70% ethanol and placed in the shake-flask.

## Results

### L-Carnosine Decreases the Specific Growth Rate of Yeast Cells Grown on the Fermentable Carbon Source, 2% Glucose

When grown in liquid culture on a fermentable carbon source such as 2% glucose, yeast cells exhibit a characteristic growth profile. During exponential growth, respiration is inhibited (the Crabtree effect). This is followed by a diauxic shift, after which the ethanol generated in the glucose consumption phase is metabolized aerobically. Table 1 shows that L-carnosine addition to glucose-grown yeast cultures (in 2×CBS medium) decreased their specific growth rate in a dose-dependent manner, slowing growth to 65% of the control value at a concentration of 30 mM; the growth rates of cultures containing 20 mM and 30 mM L-carnosine were calculated at the beginning of the logarithmic growth phase as the growth rates were so slow, consequently all subsequent experiments were performed in the presence of 10 mM L-carnosine in YP medium. The reduction in growth rate was consistent with a decreased rate of glucose consumption and decreased ethanol production and consumption rates, compared with control cultures (data not shown). Following the diauxic shift, L-carnosine-treated cells did not reach the same optical densities as untreated control cells, even though similar levels of ethanol were produced (data not shown). In contrast, yeast grown in the presence of 10 mM D-carnosine or in the presence of 10 mM β-alanine and/or L-histidine did not exhibit a reduced growth rate. On addition of 30 mM β-alanine and 30 mM L-histidine, there was a slowing of growth comparable to that caused by addition of 10 mM L-carnosine, but no cell death.

**Table 1:** The effect of L-carnosine, D-carnosine, L-histidine and β-alanine on the specific growth rate and viability of yeast cultures grown on fermentable (glucose) and non-fermentable (glycerol) carbon sources.

	μ (h^−1^)		Viability (%)	
L-Carnosine, D-carnosine, L-histidine or β-alanine concentrationadded to the indicated growth medium	*+2% glucose (2×CBS)*	*+2% glycerol (YP)*	*+2% glucose (2×CBS)*	*+2% glycerol (YP)*
Control (0 mM L-carnosine); n = 3	0.41 (0.00)	0.20 (0.01)	99.56 (0.12)	97.60 (1.04)
10 mM L-carnosine; n = 3	0.36** (0.01)	0.22* (0.01)	90.09* (0.28)	99.14 (0.63)
20 mM L-carnosine; n = 5	0.28** (0.01)	–	86.31** (0.94)	–
30 mM L-carnosine; n = 5	0.27** (0.02)	0.25** (0.01)	82.71** (1.67)	97.52 (1.92)
10 mM D-carnosine; n = 3	0.44 (0.00)	0.30** (0.00)	99.35 (0.33)	97.31 (0.34)
10 mM L-histidine; n = 6	0.40 (0.02)	–	99.94 (0.04)	–
10 mM β-alanine; n = 3	0.41 (0.00)	–	99.78 (0.12)	–
10 mM L-histidine, 10 mM β-alanine; n = 2	0.42 (0.00)	–	100 (0.00)	–
30 mM L-histidine, 30 mM β-alanine; n = 3	0.37** (0.01)	0.23* (0.00)	99.81 (0.24)	98.45 (1.55)

Yeast cells were grown in shake-flasks at 30°C in 2×CBS medium supplemented with 2% glucose or in YP supplemented with 2% glycerol as well as L-carnosine, D-carnosine, L-histidine or β-alanine (0–30 mM). Replicate cultures were performed as indicated and specific growth rates (μ; derived from OD_600_ growth curves) and viability (%; determined by trypan blue dye exclusion) were determined; a dash (–) indicates that the experimental condition indicated was not investigated. Data were analyzed using a one-way ANOVA (P<0.0001). Asterisks show the significance of the specific growth rate data for each culture condition compared to the control, as determined by a Dunnett’s multiple comparison test, where * = P≤0.05 and ** = P≤0.01. The standard error of the mean (SEM) is given in parentheses.

### L-Carnosine Reduces the Viability of Glucose-Grown Cells

**Figure 1:**
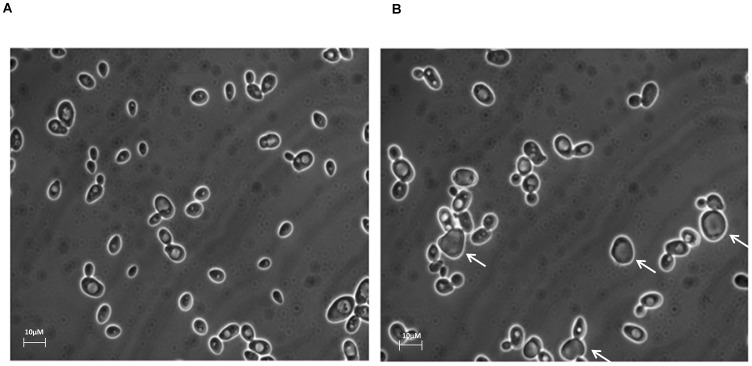
L-Carnosine addition alters the morphology of yeast cells grown on the fermentable carbon source, 2% glucose. Yeast cells cultured in shake-flasks at 30°C in 2×CBS medium supplemented with 2% glucose (A) were compared with those where the growth medium also contained 10 mM L-carnosine (B). Cells were visualized by light microscopy after reaching stationary phase, 48 h post inoculation. A proportion of the L-carnosine-treated cells (indicated with a white arrow; B) appear larger and more irregular in shape than control cells. The bar is 10 µM in length.

Microscopic examination of the yeast cultures revealed visual differences between L-carnosine-treated and control cells (Figure 1). On average, the longest dimension of L-carnosine-treated cells was 3.4% longer than control cells (n = 100). When assayed for viability by trypan blue dye exclusion, L-carnosine addition was observed to cause an increase in the proportion of blue-stained cells (an indicator of cell death) during culture growth, varying from 10% in 10 mM L-carnosine to 17% in 30 mM L-carnosine (Table 1). Notably, yeast cells with a changed morphology were not necessarily stained blue. Yeast grown in 10 mM D-carnosine or in 10–30 mM β-alanine or/and L-histidine did not exhibit reduced viability compared with control cells (Table 1).

### L-Carnosine Affects the Metabolism of Glucose-grown Cells

**Figure 2:**
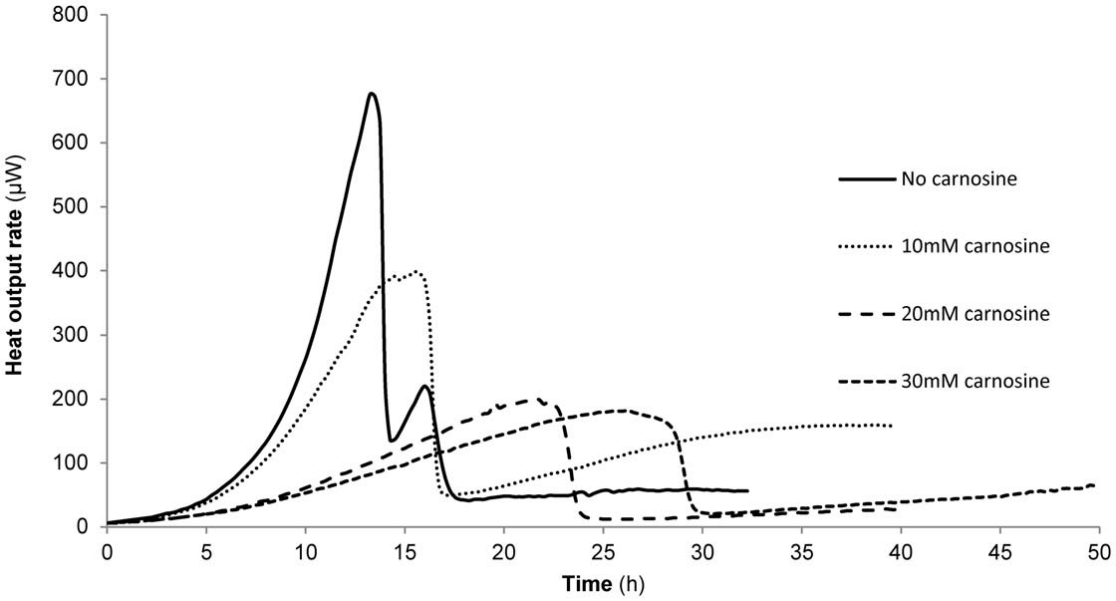
L-Carnosine addition profoundly affects the metabolism of yeast cells grown on the fermentable carbon source, 2% glucose. Yeast cells were cultured in shake-flasks at 30°C in 2×CBS medium supplemented with 2% glucose in the presence of 0–30 mM L-carnosine, as indicated, and the heat output rate measured by on-line flow microcalorimetry. Control cells exhibit a classic biphasic metabolic profile on glucose; on addition of L-carnosine the profile is altered and the delayed onset of a single, broad peak is observed.

Since L-carnosine addition reduced the specific growth rate derived from growth curves of glucose-grown cells (Table 1), on-line flow microcalorimetry was used to assess whether this was accompanied by changes in metabolic activity [18]. Figure 2, shows that this was indeed the case, with L-carnosine addition profoundly affecting the heat output profiles of the cultures in a dose-dependent manner; with increasing L-carnosine concentrations the profiles become monophasic in nature and the peaks are more delayed and broader compared to the control profile. The trend in pseudo first order rate constants calculated from the glucose phase of the microcalorimetry traces [18] mirrored that of the specific growth rates in Table 1, being 0.37 h**^−^** ^1^ (0.00; n = 4) for control cells with a dose-dependent reduction to 0.28 h**^−^** ^1^ (0.02; n = 4), 0.21 h**^−^** ^1^ (0.03; n = 2) and 0.14 h**^−^** ^1^ (0.02; n = 2) for cells grown in the presence of 10, 20 and 30 mM L-carnosine, respectively. Even when addition of the L-carnosine was delayed until mid-exponential growth, the resultant heat output rate began to resemble that of cells grown initially in L-carnosine (data not shown), suggesting that L-carnosine exerts its effects immediately. Close examination of the microcalorimetry traces revealed that in the presence of L-carnosine, growth did not fit strictly to an exponential curve prior to the diauxic shift. Together with the observation that L-carnosine addition causes a subset of yeast cells to die in culture (Table 1), this might explain the numerical differences between the two sets of rate data; the OD_600_ growth curves will necessarily yield higher growth rates than the heat output curves as the former measures the density of all cells, whilst the latter only measures metabolically active ones.

### L-Carnosine Increases the Specific Growth Rate of Cells Grown on the Non-fermentable Carbon Source, 2% Glycerol, and does not Reduce their Viability

**Figure 3:**
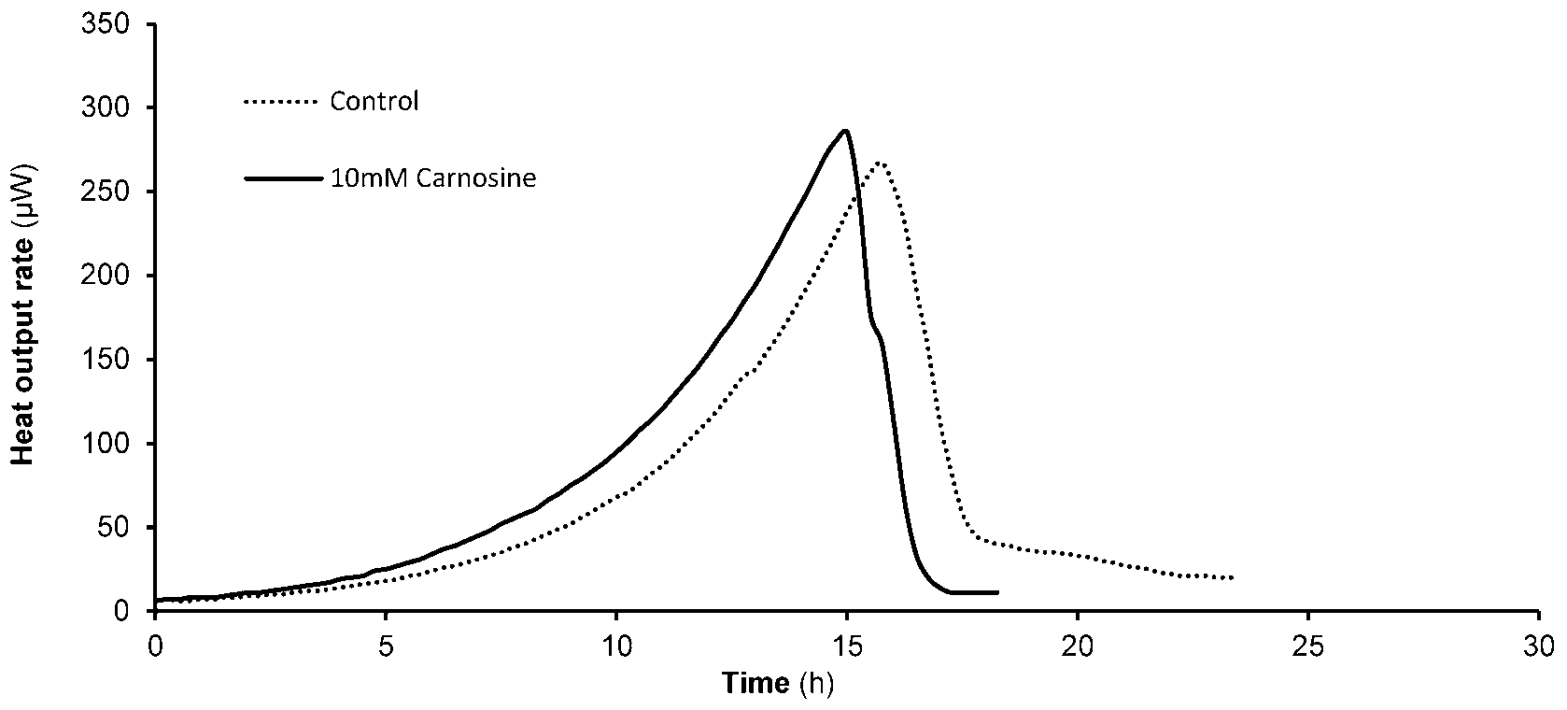
L-Carnosine addition affects the heat output rate of yeast cells grown on the non-fermentable carbon source, 2% glycerol. Yeast cells were cultured in shake-flasks at 30°C in YP supplemented with 2% glycerol in the presence of 0–30 mM L-carnosine, as indicated, and the heat output rate measured by on-line flow microcalorimetry. Control cells exhibit a classic monophasic metabolic profile on glycerol; on addition of L-carnosine the profile is altered only slightly as the onset of the peak is earlier.

When 10–30 mM L-carnosine was added to YP growth medium containing a non-fermentable carbon source (2% glycerol) there was no inhibitory effect on the growth of yeast cells. Instead, the dipeptide provoked an increase in the specific growth rates of glycerol-grown cells by up to 25% (Table 1). Consistent with this, there was no decrease in cell viability, as determined by trypan blue dye exclusion (Table 1). The effects of D-carnosine, β-alanine and L-histidine were also examined. When D-carnosine was added at a concentration of 10 mM or when both β-alanine and L-histidine were added at a concentration of 30 mM, they caused an increase in specific growth rate over control cells and did not decrease viability (Table 1). Notably, addition of D- carnosine provoked a 50% increase in growth rate compared to control cells. Microcalorimetry traces showed no statistically significant difference in metabolic activity, but the pseudo first order rate constants reflected the growth rate data in Table 1, being 0.23 h**^−^** ^1^ (0.16; n = 2) for control cells and 0.29 h**^−^** ^1^ (0.15; n = 4) for cells grown in the presence of 10 mM L-carnosine (Figure 3).

### The Metabolism-dependent Effects of L-carnosine are Observed for Cells Grown on a Range of Carbon Sources

A screen in YP medium for the effect of L-carnosine on cells cultured on the fermentable carbon sources, mannose, galactose and fructose, and the non-fermentable carbon sources, xylose, ribose and sorbitol, was performed. Table 2 shows that cells grown on all fermentable carbon sources tested experienced a reduction in viability in the presence of L-carnosine and that the decrease in viability was smaller than for cells cultured on glucose. This may reflect the fact that these carbon sources do not repress respiration to the same extent as glucose [15]. Cells cultured on the non-fermentable carbon sources behaved similarly to those cultured on glycerol, possibly exhibiting increased viability in the presence of 10 mM L-carnosine.

**Table 2:** The effect of L-carnosine on the viability of yeast cultures grown on other fermentable and non-fermentable carbon sources.

	Viability (%)	
Carbon source added to YP medium (2%)	*no L-carnosine*	*+10 mM L-carnosine*
***Fermentable carbon sources***		
Glucose	99.33 (0.33)	84.85 (1.89)
Mannose	100 (0.00)	97.83 (0.56)
Galactose	99.67 (0.33)	96.85 (0.56)
Fructose	99.38 (0.62)	97.68 (1.26)
***Non-fermentable carbon sources***		
Glycerol	97.56 (1.04)	99.14 (0.63)
Xylose	99.35 (0.32)	99.69 (0.31)
Ribose	99.35 (0.33)	100 (0.00)
Sorbitol	99.08 (0.53)	100 (0.00)

Yeast cells were grown in shake-flasks at 30°C in YP medium supplemented with the indicated carbon source with or without 10 mM L-carnosine. Viability (%; determined by trypan blue dye exclusion) was determined in triplicate. The standard error of the mean (SEM) is given in parentheses.

### The Respiratory Yeast, Pichia pastoris, is Resistant to L-carnosine-induced Cell Death

The yeast species *Pichia pastoris,* is a Crabtree-negative yeast as it exhibits a respiratory metabolism when glucose is abundant [19]. Addition of 10 mM L-carnosine to cultures of *P. pastoris* was therefore examined to confirm the dipeptide’s metabolism-dependent effects. In *P. pastoris* cultures, viability was 99.35% (SEM; 0.65) for untreated cells and 99.67% (0.33) in the presence of 10 mM L-carnosine and 2% glucose.

### Yeast Strains with Deletions in Nutrient-sensing Pathways are Resistant to L-carnosine-induced Cell Death

Nutrient-sensing pathways link nutrient availability with cell growth and metabolism. In yeast, these nutrient-sensing pathways include the target of rapamycin (TOR), Sch9 protein kinase and protein kinase A (PKA) pathways [20]. Yeast strains with deletions in these pathways are often referred to as calorie restriction mimics, as down-regulation of the corresponding kinases limits excess nutrient intake and promotes longevity in yeast [20]. The down-regulation of these pathways also causes an increased dependence on a respiratory metabolism [21], [22], [23], [24]. A selection of deletion strains was therefore chosen to investigate the metabolism-dependent effects of L-carnosine addition to yeast cells (Table 3). In a *tor1Δ* strain, an increase in oxygen consumption, mitochondrial translation and proteins associated with respiration is observed [24]; Rpl31a is a downstream protein target of the TOR pathway [20]. As part of the PKA pathway, the G protein-coupled receptor, Gpr1, activates the alpha subunit Gpa2; activation of PKA (which comprises 3 functionally redundant catalytic subunits, Tpk1-3, and a regulatory subunit Bcy1) in response to nutrient availability also causes an increase in the expression of genes associated with fermentation. Down-regulation of this pathway is associated with an increase in respiration and a decrease in fermentation [23]. The Sch9 pathway has very similar functions: deletion of pathway components causes an increased number of mitochondria, increased oxygen consumption and an increase in proteins involved in respiration [21]. Hxk2, is a hexose kinase involved in the phosphorylation of glucose to glucose-6-phosphate. The *hxk2*Δ strain has respiratory characteristics [25].

**Table 3:** The effect of L-carnosine on the viability of yeast strains with deletions in the TOR, Sch9 and PKA nutrient sensing pathways.

	Viability (%)	
Strain	*no L-carnosine*	*+10 mM L-carnosine*
Wild-type	99.33 (0.33)	84.85 (1.89)
*tor1*Δ	99.87 (0.20)	94.20 (1.30)
*rpl31*Δ	100 (0.00)	99.33 (0.67)
*gpa2*Δ	100 (0.00)	97.99 (0.22)
*tpk1*Δ	100 (0.00)	91.67 (2.25)
*gpr1*Δ	100 (0.00)	97.42 (1.21)
*sch9*Δ	100 (0.00)	96.44 (0.33)
*hxk2*Δ	100 (0.00)	98.22 (1.07)

Yeast cells were grown in shake-flasks at 30°C in YP medium supplemented with 2% glucose, with or without 10 mM L-carnosine. Viability (%; determined by trypan blue dye exclusion) was determined in triplicate. The standard error of the mean (SEM) is given in parentheses.

Table 3 shows that these deletion strains were resistant to L-carnosine-induced cell death compared with wild-type cells, with the largest reduction in viability being seen for *tpk1*Δ, as might be expected on account of its functional redundancy.

## Discussion

L-Carnosine causes an increase in the lifespan of human fibroblasts [3] and *E. coli* cells [26]. Our experiments to determine the effect of L-carnosine on yeast revealed a profound effect on the viability of glucose-grown cells (Table 1; Figure 2), consistent with earlier findings by Holliday and McFarland, who showed that L-carnosine inhibits the growth of cancer cells [7]. More recently, in an attempt to understand the mechanisms responsible for L-carnosine-induced inhibition of cancer cell growth, two studies have shown that L-carnosine addition to tumor cells results in a reduction in cellular ATP production [9], [27] from glycolysis [9].

When *S. cerevisiae* is grown on a fermentable carbon source, the Crabtree effect [15] leads to a decrease in oxidative phosphorylation. Addition of L-carnosine promotes cell death under these conditions (Table 1), as also seen for cancer cells [7]. The fermentable carbon sources, mannose, galactose and fructose, also repress oxidative phosphorylation, but to a lesser degree than glucose [15], consistent with the smaller L-carnosine-induced reduction in the viability of yeast cultures grown on these carbon sources (Table 2).

To explore whether a shift of metabolic flux to fermentation could provide mechanistic insight into how L-carnosine influences viability in yeast cells, the consequence of L-carnosine addition to yeast cultures grown on the non-fermentable carbon sources, glycerol, xylose, ribose and sorbitol was explored (Tables 1, 2); non-fermentable carbon sources require the presence of oxygen to be metabolized via oxidative phosphorylation. Table 1 shows that L-carnosine addition does not reduce the viability of these yeast cultures. An increased dependence on respiration, similar to the effect on non-fermentable carbon sources, may underlie these results, as seen for the respiratory yeast species, *P. pastoris*, which is also minimally affected by L-carnosine addition. We therefore propose that L-carnosine has a detrimental effect on cells when their energy generation is dependent on glycolysis.

Closer examination of our yeast optical density (Table 1, 2) and microcalorimetry rate data (Figure 3) shows that L-carnosine may have a tendency to promote cell growth on glycerol, possibly by acting as an amino acid source. The slightly improved viability of yeast grown on a non-fermentable carbon source following L-carnosine addition could also reflect the anti-senescence properties seen in human fibroblasts when grown in the presence of L-carnosine, i.e. decreased glycolysis and increased mitochondrial activity [3], [28].

The mechanisms by which L-carnosine exerts its inhibitory effects on growth and viability remain uncertain, one proposal being the ATP-lowering effect mentioned above [9], [27]. Early work demonstrated that L-carnosine stimulated the activity of the gluconeogenic enzyme fructose-1-6-biphosphatase [29] which converts fructose-1-6-bisphosphate to fructose-6-phosphate. Should L-carnosine stimulate fructose-1-6-bisphosphatase in yeast, an ATP-consuming futile cycle might be created in glucose-grown cells, but not in cells growing on glycerol. By limiting the availability of fructose-1-6-bisphosphate for conversion by aldolase to triose-phosphates, both ATP generation and the supply of precursors for macromolecule synthesis would be reduced. Our data for deletion mutants in nutrient sensing pathways (Table 3), which have an increased dependence on a respiratory metabolism [21], [22], [23], [24] and hence exhibit a decreased response to L-carnosine addition, would fit with this hypothesis.

Overall, our data for yeast cells grown on glucose are consistent with observations previously published for cancer cells [7], [8], [9]. Furthermore studies with yeast cells have an additional advantage in that the action of L-carnosine can be examined under aerobic growth on glycerol; in this study we show that L-carnosine adversely affects fermentative growth on glucose, while aerobic growth on glycerol is not inhibited. In contrast, D-carnosine does not affect growth on glucose, but significantly stimulates growth on glycerol, suggesting these metabolism-dependent effects are mediated by different mechanisms. Yeast cells therefore provide an amenable system whereby the metabolism-dependent effects of L-carnosine are tractable for further study.
